# Patterns associated with hunting with dogs in a semiarid region of northeastern Brazil

**DOI:** 10.1186/s13002-022-00570-4

**Published:** 2022-12-18

**Authors:** Sebastiana Lima Santos, María Fernanda De la Fuente, Rômulo Romeu Nóbrega Alves

**Affiliations:** 1 Programa de Pós-Graduação em Ecologia e Conservação, Departamento de Biologia, Universidade Estadual da Paraíba, Campina Grande, PB Brazil; 2 Programa de Pós-Graduação em Etnobiologia e Conservação da Natureza, Universidade Estadual da Paraíba, Campina Grande, PB 58019-753 Brazil; 3 Departamento de Biologia, Universidade Estadual da Paraíba, Campina Grande, PB Brazil

**Keywords:** *Canis lupus familiaris*, Ethnozoology, Hunting dog, Caatinga, Wildlife conservation

## Abstract

**Background:**

Hunting has been an important cultural and subsistence activity for the survival of the human population. In the Brazilian semiarid region (Caatinga), the extreme seasonal changes and socioeconomic conditions have made local people dependent on the natural resources available, including wildlife. Although hunting with dogs can result in higher efficiency for hunters, it can also have implications for game species conservation.

**Methods:**

Using an ethnozoological approach (semi-structured questionnaires, free interviews, informal conversations, and free listing technique), this study aimed to analyze the patterns of hunting with dogs activities in a semiarid region of northeastern Brazil by characterizing hunters’ and hunting dogs’ profiles, investigating target and nontarget prey species, hunters’ practices, motivations, and perceptions regarding the efficiency of hunting with dogs.

**Results:**

We found that hunters that use dog assistance were mostly men, of different ages, with an occupation in agriculture, receiving less than a minimum wage, and with a low level of formal education. Hunters use two or more mixed-breed dogs with no clear preference regarding dogs’ sex. The motivations for hunting with dogs included mainly food, sport, and trade. Hunters cited twenty species captured by dogs without distinction between prey’s sex and age (14 mammals, 4 birds, and 2 reptiles). Only six of these were mentioned as being target prey when hunting with dogs. From nontarget species, eight carnivores are usually left at the site of kill, as they have no use to the hunters. Hunters perceived that hunting with dogs could be three times more efficient than hunting without dogs.

**Conclusion:**

Overall, hunting with dogs represents a complex set of local variables, including characteristics of dogs and prey species, hunters’ motivations, and practices that should be considered according to each particular situation. Considering the human dependence on natural resources in the semiarid region, hunters should be included in wildlife management debates to mitigate the threat to game species while allowing sustainable hunting practices.

## Introduction

Hunting has been an important activity for the survival of the human population throughout the history of humanity [1]. Until the present time, wild animal resources are exploited for several uses, such as food procurement, protection against weather (clothing using leather and skin), defense against wild predators, medical applications, tool making, and magic-religious purposes [2–9]. Other uses like keeping animals as pets, the trade of animals and their products for different intentions, and recreational or sport hunting have also motivated this practice [1]. Throughout the tropics and subtropics, bushmeat is an important component of rural livelihoods primarily for subsistence (own consumption), while contributes to households’ income (generated through trade) less than previously thought [10–14]. In Brazil, the Environmental Criminal Law (N° 9605/1998, article 37) legalizes hunting for subsistence purposes. In indigenous and traditional communities, as well as rural and urban populations characterized by extreme poverty, such practice constitutes an important livelihood factor and the main or only source of protein in their diet [15].

The Caatinga, a semiarid region of northeast Brazil, is a seasonally dry forest receiving less than 500 mm of rain per year at many sites [16]. It has a climate classified as BSh type according to the Köppen climate classification (hot semiarid (steppe) climate; [17]), characterized by high temperatures, low humidity, and extended periods of severe droughts (3 to 6 months a year) [16]. In addition, Caatinga is one of the poorest regions in Brazil [18]. The extreme seasonal changes and socioeconomic conditions have made people living in this region depend on the natural resources available in the environment. For example, during long periods of drought that damage crops, the body condition of domestic animals is poor due to limited water and food availability, game meat becomes a source of food, while the trade of other animal parts, such as skin and leather, provides a supplementary family income [19–21]. From a cultural perspective, even when there are alternative sources of food/protein and income in some communities, hunting also plays an important social role in people’s livelihood, as an entertainment and recreational practice [1].

Hunters must possess and retain detailed ecological knowledge about prey species’ habits and location to successfully perform hunting activities. Different hunting techniques and strategies have been developed to improve hunting competence in the semiarid region [20, 22]. Hunters usually use more than one technique depending on the target prey type, behavior, availability, accessibility, environment, and intended use. Passive hunting strategies do not require the presence of the hunter. In this type of strategy, mechanical traps (lethal or nonlethal) are set up and checked after some time, saving the hunter’s time and energy [20, 23]. On the other hand, active hunting strategies require the active presence of the hunter and search for game species. For example, the waiting or ambush game, in which hunters hide and wait in ambush for the prey, and the persistence hunting, in which hunters pursue the prey until its exhaustion. In addition, hunting with the support of accessories such as tools (e.g., weapons, shotguns, firearms) and other animals (e.g., dogs, falcons) can bring greater efficiency to hunting activities [1, 20].

In some regions, hunting constitutes a major challenge to biodiversity conservation [24]. The improvement in hunting technologies and the commercialization of hunting can represent a threat to wildlife, especially for overhunted species, resulting in rapid forest defaunation, potentially leading to what is known as “empty forest” (i.e., extinction or ecological extinction of animal species in forests where the vegetation appears intact; 25, 26). Because dogs can enhance the hunter’s and hunting efficiency, hunting with dogs can potentially have a greater impact on wildlife than other forms of hunting without dogs, leading to the reduction in local game populations and resulting in conservation concerns [24]. Therefore, understanding the patterns associated with hunting with dogs and its implication for wildlife is crucial to developing management measures and improving conservation strategies in the region, taking into account the needs of local communities. Here, we aimed to understand the patterns of hunting with dogs activities in a semiarid region of northeastern Brazil. Specifically, we characterized hunters’ and their hunting dogs’ profiles, investigated which prey species (target and nontarget) are hunted using this technique as well as their motivation of use, and examined hunters’ perceptions regarding the efficiency of hunting with dogs versus hunting without dogs. Considering the socioeconomic conditions of the Caatinga, we hypothesize that (1) hunters are mainly men, of different ages, who work in agricultural activities, have a low income and a low educational level; (2) hunting dogs are mostly mixed-breed; (3) the motivations for hunting with dogs are related to food and recreation; (4) hunters perceive a greater hunting efficiency when hunting with dogs than when hunting without dogs. Finally, we discussed the implications of this activity for game species conservation.

## Methods

### Study area

**Fig. 1:**
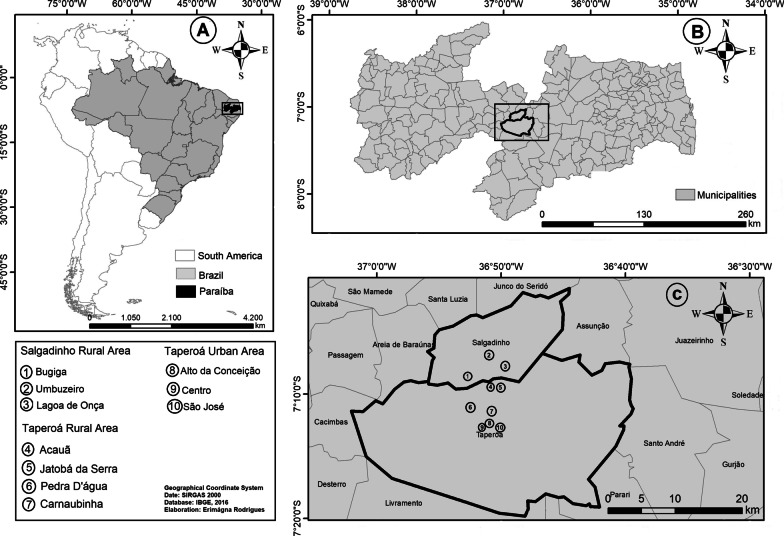
Location of the Taperoá and Salgadinho study communities (**C**), in the state of Paraíba (**B**), Brazil (**A**)

The study was carried out in the Caatinga semiarid region of northeast Brazil, specifically in the municipalities of Taperoá (7° 12′ 28″ S, 36° 49′ 34″ W) and Salgadinho (7° 48′ 0″ S, 36° 34′ 60″ W) located in the central region of the state of Paraíba, inserted in the Borborema mesoregion. For both municipalities, the vegetation is predominantly low with low arboreal shrubs density, typical of this semiarid region [27]. The annual average temperature and rainfall are 24 °C (range from 21 to 28 °C) and 505.6 mm (range from 500 to 750 mm), respectively [28, 29]. Taperoá has an area of approximately 62,800 ha with an estimated population of ~ 15,500 inhabitants from urban (~ 9300 people) and rural areas (~ 6200 people) and Salgadinho has an area of approximately 18,400 ha with an estimated population of ~ 3.975 inhabitants, from urban (~ 1.354 people) and rural areas (~ 2.621 people) [30]. In Taperoá, the study was conducted in three neighborhoods from the urban area (Alto da Conceição, Centro, and São José) and in four communities from the rural area (Acauã, Jatobá da Serra, Pedra D'água, and Carnaubinha). In Salgadinho, the study was conducted in three communities from the rural area (Bugiga, Umbuzeiro, and Lagoa de Onça) (Fig. 1).

### Data collection

The research was conducted from October 2018 to May 2019. Informant selection was performed through the snowball sampling technique [31], intentionally selecting those people who hunted with the help of dogs. The key informants (more experienced hunters) were selected by the criterion of “local experts” who were recognized by the community as culturally competent [32]. These experts indicated other hunters and, in total, 47 local hunters (11 from Taperoá urban area, 12 from Taperoá rural area, and 24 from Salgadinho rural area) voluntarily agreed to participate in the survey. Information was gathered through semi-structured questionnaires, free interviews, and informal conversations [33]. To seek reliable answers from the interviewees, the interviewer sought to initiate a pleasant dialog, involving topics such as interactions and affection with their dog, the pleasure of hunting, and contact with nature. As the informal conversation became more relaxed, specific hunting questions were introduced. Monthly contact with informants was made to maintain trust between the interviewees and the interviewer. The interviewer (the first author) has a preexisting relationship with the community, which facilitated the dialog.

During the interviews, specific information was obtained on hunters’ socioeconomic profiles (gender, age, occupation, monthly income, education), hunting dogs’ profiles (preference for dog’s breed and sex, number of dogs used, their training and maintenance), hunting dogs’ keeping practices (housing, feeding, hygienic care, monthly expenses, health), questions related with the practice of hunting with dogs (frequency, motivation, hunting period, association with firearms), species hunted by dogs (target and nontarget prey), and hunters’ perceptions regarding the efficiency of hunting with dogs (number of specimens hunted per expedition with and without dogs, most hunted species, hardest species to be found by dogs, prey abundance in the region). The free listing technique was used to identify the species captured by dogs during expeditions. To verify the conservation status of the recorded species, the Brazilian List of Fauna Threatened with Extinction [34] and the International Union for Conservation of Nature Red List of Threatened Species [35] were used.

### Data analysis

Descriptive statistics were used to describe patterns related to hunters’ socioeconomic profiles, hunting dogs’ profiles, the frequency of hunting with dogs, hunting motivation, hunting period, and association with firearms. From the free lists of species cited to be captured with the help of dogs, the salience of species was calculated using the Smith’s Salience Index [36]. The salience represents a measure of the cultural importance of the items belonging to a domain. It ranges from 0 to 1 and is expressed by the relation between the frequency of citations and the order of citations of each item, allowing the ordering of items from most salient (values near 1) to least salient (values near to 0) item.

To test hypotheses 1, 2, and 3, chi-square goodness-of-fit analyses were performed to compare the observed frequencies of (1) hunters’ socioeconomic characteristics (gender, age categories, occupation, monthly income, and educational level); (2) hunting dog’s breed; and (3) the declared motivations for hunting with dogs, versus expected frequencies (i.e., frequencies in each category of categorical variables are equal: null hypothesis). The age variable was divided into five categories (up to 29 years, 30–39 years, 40–49 years, 50–59 years, and more than 60 years). The occupation variable was divided into two categories (agricultural and nonagricultural activities). The monthly income variable was divided into three categories, taking into account hunters’ responses (less than the Brazilian minimum wage, one to two times the minimum wage, and two to three times the minimum wage). The educational level variable was divided into five categories according to hunters’ responses (did not attend school, incomplete elementary school, complete high school, incomplete higher education, and complete higher education). The hunting dogs’ breed variable was divided into mixed-breed and breed dogs. Finally, the motivation for hunting with dogs variable was divided into three categories (food/flavor, sport/pleasure, and trade).

To estimate the informants’ perceptions and investigate the difference in efficiency perceived of hunting with and without dogs (hypothesis 4), the informants were asked to indicate the mean number of target specimens hunted with the help of dogs and the mean number of the same target specimens hunted without dogs (e.g., tracking or using traps) per expedition. Shapiro–Wilk analyses were used to test data normality. Since data were not normal, we used Wilcoxon signed-rank test (paired data) to compare hunters’ perceptions of hunting with and without dogs. All analyses were conducted using R studio software, version 3.6.2 [37]. For all analyses, the statistical significance level was set at 0.05.

## Results

### Hunters’ socioeconomic profile

**Fig. 2:**
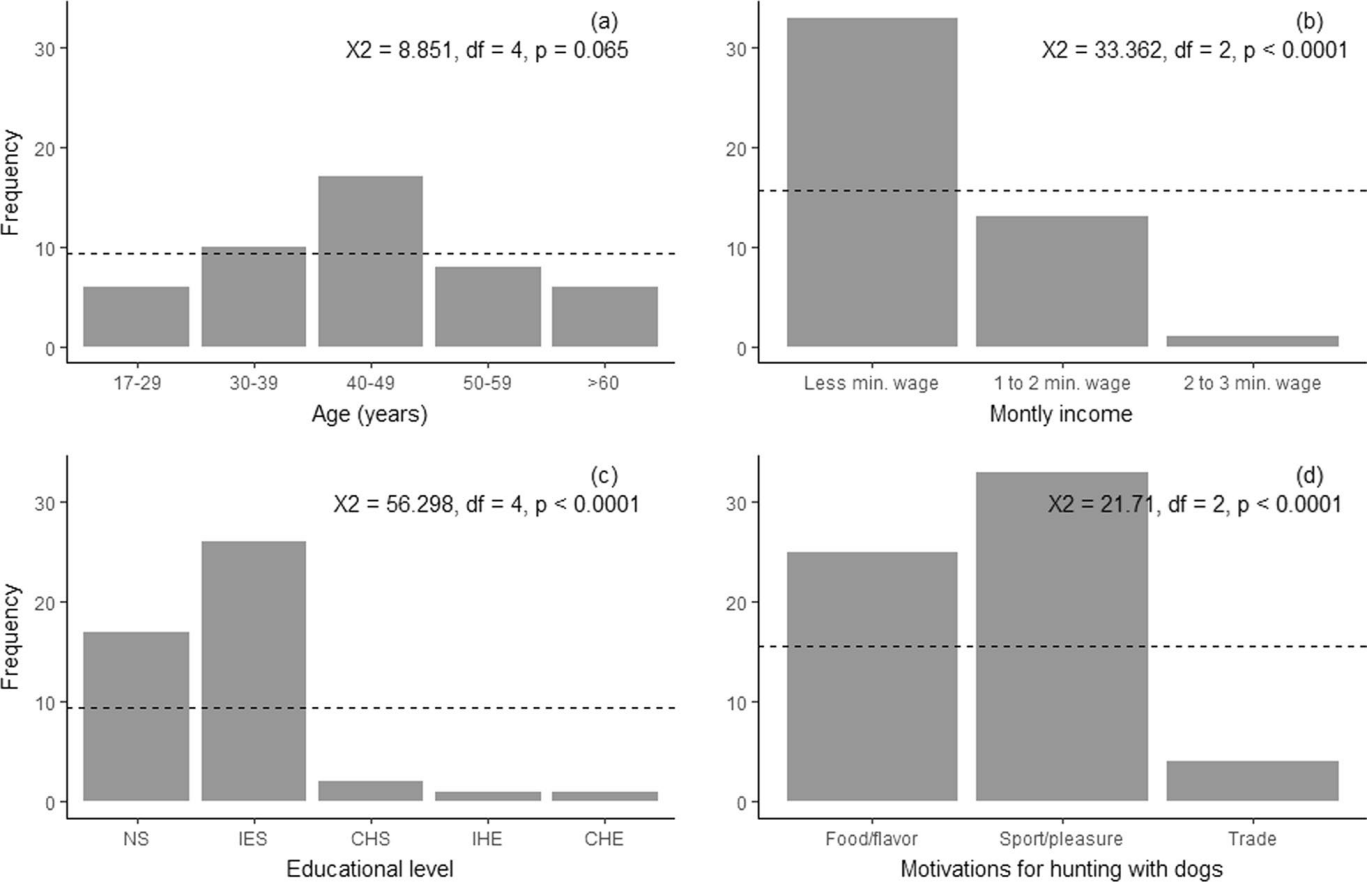
Observed frequencies of hunters’ **a** age, **b** monthly income, **c** educational level, and **d** motivations for hunting with dogs. Dashed lines indicate the expected frequency values for each variable (all categories are equal: null hypothesis). NS: No school; IES: Incomplete elementary school; CHS: Complete high school; IHE: Incomplete higher education; CHE: Complete higher education

Of the 47 hunters that hunted with dog assistance, 43 (91.5%) were men and 4 (8.5%) were women (*X*^2^ = 32.362, *df* = 1, *p* < 0.0001). Their ages ranged from 17 to 82 (mean ± SD = 44.6 ± 15.1 years) (*X*^2^ = 8.851, *df* = 4, *p* = 0.065, Fig. 2a). Interviewees’ occupations were mainly agriculture (*N* = 34, 72.3%) and others (*N* = 13, 27.7%) including mason, social worker, school agent, and barber, among others (*X*^2^ = 9.383, *df* = 1, *p* < 0.002). More than half of the respondents (*N* = 33, 70.2%) received less than the Brazilian minimum wage (R$ 998 equivalent to ~ US$ 255), 13 respondents (27.7%) received one to two times the minimum wage, and one respondent (2.1%) received two to three times the minimum wage (*X*^2^ = 33.362, *df* = 2, *p* < 0.0001, Fig. 2b). Finally, 36.3% (*N* = 17) of the respondents declared that they did not attend school, 55.3% (*N* = 26) declared that their educational level was incomplete elementary school, 4.2% (*N* = 2) completed high school, 2.1% (*N* = 1) did not complete higher education, and 2.1% (*N* = 1) completed higher education (*X*^2^ = 56.298, *df* = 4, *p* < 0.0001, Fig. 2c).

### Hunting dogs’ profile

All interviewed hunters hunted using mixed-breed dogs; only four of them also mentioned the use of Pointer (“perdigueiro” in Portuguese) (*X*^2^ = 36.255, *df* = 1, *p* < 0.0001). When asked why they preferred these breeds, respondents answered that mixed-breed dogs capture any wild species, are better adapted to the region and to their socioeconomic conditions (*N* = 33, 70.2%) and that they have an easier time learning, better resistance, agility, intelligence, and experience (*N* = 14, 29.8%). Pointers are used in diurnal expeditions to capture *Crypturellus* sp., in which the dog sniffs and scares the bird away so that the hunter can target it with a firearm and later the dog brings the downed bird.

Regarding dogs´ sex, 14 respondents (29.8%) did not have a sex preference and hunted with both male and female dogs, 15 respondents (31.9%) used only females, and 18 respondents (38.3%) used only males. Hunters that preferred females answered: this was because they are “smarter,” “calmer,” “obedient,” “have puppies,” and “learn to hunt with their mother.” While hunters that preferred males do so because they “hunt a higher quantity of specimens,” “do not reproduce” (i.e., in terms of taking care of the offspring), “females are more playful and have more difficulty concentrating,” “males are better hunters, do not get distracted,” and “are more focused.” The majority of respondents (*N* = 37, 78.7%) hunted with two dogs, only 4 of them (8.5%) used one dog, and 6 respondents (12.8%) answered that they used three dogs for hunting. Most hunters (*N* = 32, 68.1%) trained their own dogs for hunting, while 8 of them (17%) bought trained dogs at prices ranging from R$50 to R$1.500 (US$ 10,41 to US$ 312,40), and 5 hunters (10.6%) reported that had both trained and bought hunting dogs. Two hunters (4.3%) did not answer this question.

Most hunters (*N* = 43, 91.5%) reported that they kept their dog(s) restricted in their household and only 4 (8.5%) kept them unrestricted. However, 25 (53.2%) of them responded that their dog had run away into the woods to hunt by itself. In addition, twenty-six hunters (55.3%) did not take their dogs for companionship during their daily activities, while 21 (44.7%) do and 19 of them (40.4%) stated that their dogs have captured wild animals by themselves during this time (specifically, agricultural activities).

### Hunting dogs’ keeping practices

**Fig. 3:**
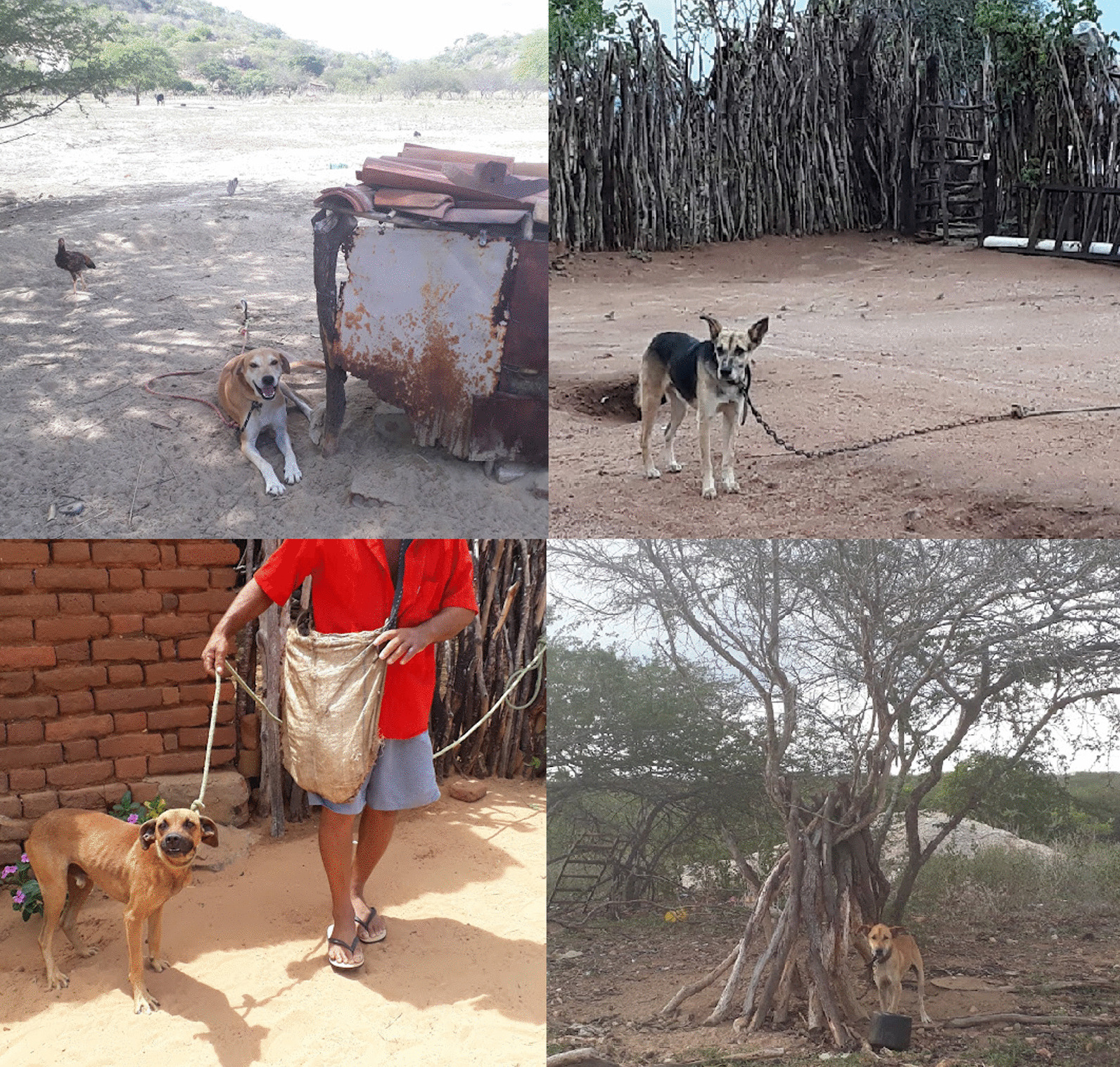
Hunting dogs from urban and rural areas of the Caatinga semiarid region of northeastern Brazil are usually kept outside (in the yard) tied up on a chain or rope

Hunters (*N* = 45) reported that they keep their dogs outside, without any type of proper doghouse, kennel or bedding. Most of them (80%, *N* = 36) keep their dogs tied up on a chain or rope under a tree (Fig. 3), while nine (20%) keep dogs loose in the fenced yard. Most hunters feed their dogs with cooked/homemade food or leftovers (84.4%, *N* = 38), while three of them (6.7%) buy dry dog food, and four (8.9%) feed their dog with both cooked/homemade food and dry dog food. Feeding frequency was reported to occur once (40%, *N* = 18) or twice a day (60%, *N* = 27). In terms of hygienic care, only seventeen hunters (37.8%) reported bathing their dog at home, on a weekly (*N* = 12), fortnightly (*N* = 1), or monthly (*N* = 4) basis. For 13 hunters (28.9%), monthly expenses with the dog ranged from R$40 to R$750 (mean = R$156.9, SD = R$187.6), while most hunters (71.1%, *N* = 32) reported not having monthly expenses with the animal. Even though all hunters answered that their dogs do not usually get sick, dog illnesses were reported by 55.6% of the hunters (*N* = 25), who mentioned 11 types of diseases or injuries, the most cited being: the presence of ticks (*N* = 17), viruses (not specified, *N* = 7), and worms (not specified, *N* = 6). Other illnesses mentioned (*N* = 1 each) were: anemia, poisoning, tremors, tick disease, urinary infection, injured paw, snake bite, and scabies. For treatments, only 13.3% of hunters (*N* = 6) took the dog to the vet, 15.6% (*N* = 7) medicated the dog without a vet indication, and 6.7% (*N* = 3) applied home treatment. Twenty percent (*N* = 9) did not answer this question. Thirty-six hunters (80%) vaccinate their dog in rabies campaigns, and 10 hunters (22.3%) reported taking their dog to the vet to be vaccinated against worms and viruses.

### Hunting with dogs

Most respondents hunted with dogs on a weekly basis (*N* = 35, 74.5%), others do so monthly (*N* = 7, 15%), daily (*N* = 4, 8.5%), or annually (*N* = 1, 2%). All hunters (*N* = 47) hunted with dogs at night, and most of them (*N* = 30) also did so during the daytime. Hunters’ indicated that they hunted species with dogs for one or several purposes, including for food/flavor (*N* = 25), for sport/pleasure (*N* = 33), and for trade (*N* = 4) (*X*^2^ = 21.71, *df* = 2, *p* < 0.0001, Fig. 2d). Most hunters (*N* = 29, 62%) responded that they do not associate firearms when hunting with dogs, while others (*N* = 18, 38%) do so to kill birds that are not usually killed by dogs, to kill other game species and/or to protect their dog against “conflict” animals (e.g., snakes and foxes).

### Species hunted by dogs

**Fig. 4:**
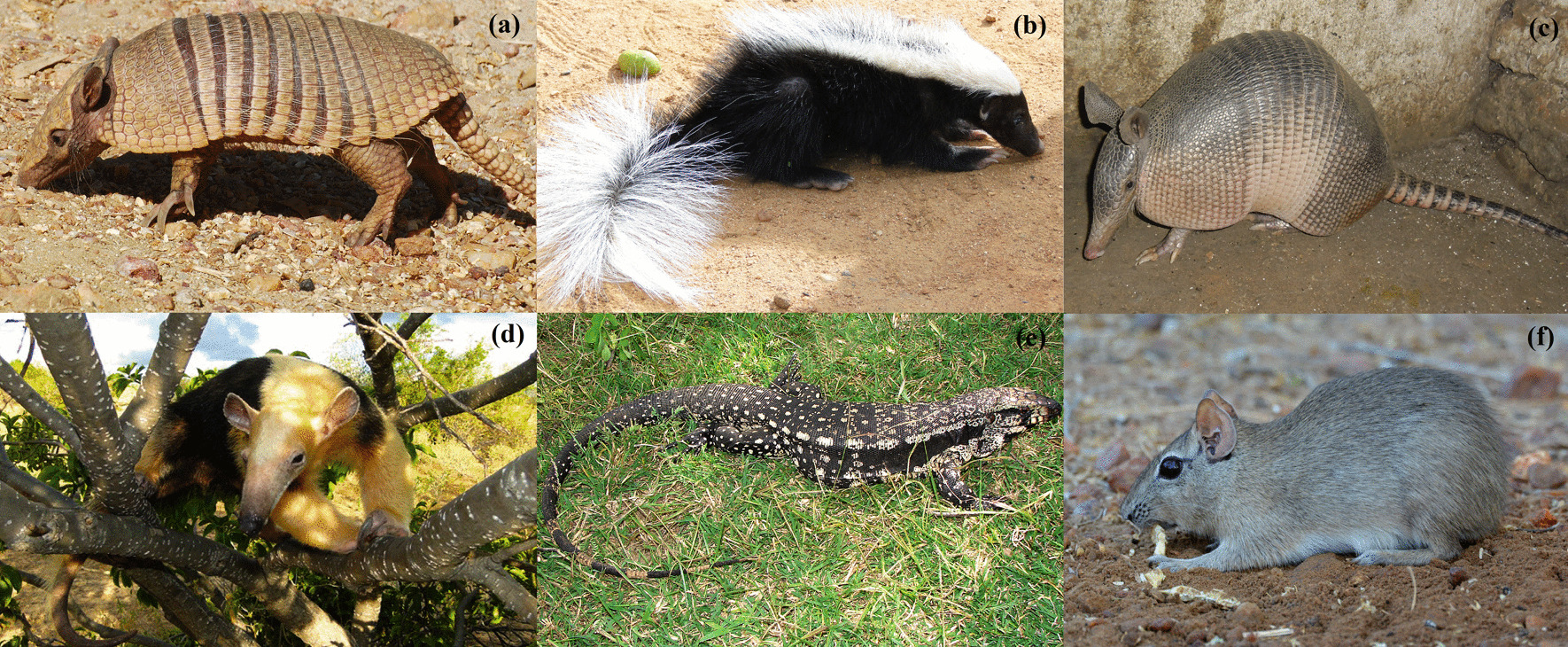
Target species when hunting with dogs according to hunters interviewed in Taperoá and Salgadinho, Paraíba, Brasil. **a** *Euphractus sexcinctus*, **b** *Conepatus semistriatus*, **c** *Dasypus novemcinctus*, **d** *Tamandua tetradactyla*, **e** *Salvator merianae*, and **f** *Galea spixii*

In the free lists, informants cited 20 species of animals, from three taxonomic groups (mammals: 14 species, birds: 4 species, reptiles: 2 species) that hunting dogs could capture during an expedition (Table 1). Of these, 6 species were mentioned as target prey when hunting with dogs, and 14 were mentioned as nontarget prey. The most salient species were the target species: *Euphractus sexcinctus* (Salience Index: 0.8981), *Conepatus semistriatus* (0.7996), *Dasypus novemcinctus* (0.7945), and *Tamandua tetradactyla* (0.5839). *Salvator merianae* (0.3846) and *Galea spixii* (0.1518) were also mentioned as target species but were less salient on the free lists (Fig. 4). All of the species mentioned above are used as food in the surveyed area, while *Conepatus semistriatus* is used both as food and for medicinal purposes (bones are crushed or cooked in broth to treat rheumatism). Hunters reported that they hunt these target species mainly as a “form of entertainment” and because they “like the taste of the meat.” When bushmeat is appreciated in terms of flavor, they gather among friends and other hunters to eat together as a form of leisure. On the contrary, when bushmeat is not appreciated (nontarget prey), they donate it to friends or relatives. Regarding trade, two hunters mentioned that, sporadically, they receive orders from local residents to use some animals as zootherapy (Table 1).

**Table 1:** Scientific name, common name in Portuguese and English, salience index, uses, hunting expedition period, and conservation status of animal species that hunting dogs can capture during an expedition as mentioned by interviewed hunters (in 47 free lists) from the communities of the municipalities of Taperoá and Salgadinho, Paraíba, Brazil

Scientific name	Common name in Portuguese	Common name in English	Salience Index	Dogs target prey	Uses	Hunting expedition period	Conservation Status IUCN/Brazilian List
*Euphractus sexcinctus* (Linnaeus, 1758)	Peba, Tatu-peba	Yellow Armadillo	0.8981	Yes	Food	Night	LC/LC
*Conepatus semistriatus* (Boddaert, 1785)	Tacaca	Striped Hog-nosed Skunk	0.7996	Yes	Food/Medicinal	Night	LC/LC
*Dasypus novemcinctus* (Linnaeus, 1758)	Verdadeiro, Tatu-galinha	Nine-banded Armadillo	0.7945	Yes	Food	Night	LC/LC
*Tamandua tetradactyla* (Linnaeus, 1758)	Tamanduá, Tamanduá-mirim	Southern Tamandua	0.5839	Yes	Food	Night	LC/LC
*Salvator merianae* (Duméril & Bibron, 1839)	Tejú, Teiú-gigante	Black-and-white Tegu	0.3846	Yes	Food/Medicinal	Day	LC/LC
*Cerdocyon thous* (Linnaeus, 1766)	Raposa, cachorro-do-mato	Crab-eating Fox	0.3352	No	None/Left at the site of kill	–	LC/LC
*Didelphis albiventris* (Lund, 1840)	Timbu, Gambá-de-orelha-branca	White-eared Opossum	0.2352	No	None/Left at the site of kill	–	LC/LC
*Herpailurus yagouaroundi* (É. Geoffroy, 1803)	Gato do mato azul/vermelho	Jaguarundi	0.1986	No	None/Left at the site of kill	–	LC/VU
*Galea spixii* (Wagler, 1831)	Preá	Spix’s Yellow-toothed Cavy	0.1518	Yes	Food	Day	LC/LC
*Procyon cancrivorus* (G. Cuvier, 1798)	Guaxinim/Guará	Crab-eating Raccoon	0.1158	No	None/Left at the site of kill	–	LC/LC
*Leopardus tigrinus* (Schreber, 1775)	Gato do mato pintado	Northern Tiger Cat	0.0835	No	None/Left at the site of kill	–	VU/EN
Undefined	Gato do mato	Undefined	0.0665	No	None/Left at the site of kill	–	-
*Galictis vittata* (Schreber, 1776)	Furao, Furao-grande	Greater Grison	0.0615	No	None/Left at the site of kill	–	LC/LC
*Iguana iguana* (Linnaeus, 1758)	Camaleao, Iguana-verde	Common Green Iguana	0.0457	No	Food	Day	LC/LC
*Crypturellus sp.*	Lambu, Inhambu	Tinamou	0.0173	No	Food	Day	LC/LC
*Cariama cristata* (Linnaeus, 1766)	Seriema	Red-legged Seriema	0.0125	No	Food	Day	LC/LC
*Dendrocygna viduata* (Linnaeus, 1766)	Pato D'água, Irerê	White-faced Whistling-duck	0.0089	No	Food	–	LC/LC
*Leopardus wiedii* (Schinz, 1821)	Gato do mato maracajá	Margay	0.0088	No	None/Left at the site of kill		NT/VU
*Kerodon rupestris* (Wied-Neuwied, 1820)	Mocó	Rock Cavy	0.0053	No	Food/Medicinal	Day	LC/VU
*Nothura boraquira* (Spix, 1825)	Codorniz, Codorna-do-nordeste	White-bellied Nothura	0.0013	No	Food	–	LC/LC

LC, least concerned; VU, vulnerable; EN, endangered; NT, near threatened

From nontarget species, all birds cited (*Crypturellus sp., Cariama cristata, Dendrocygna viduata, Nothura boraquira*) are used as food when eventually captured, and a reptile (*Salvator merianae*) is used both as food and for medicinal purposes (lard used to treat sore throat). In addition, 8 of the nontarget species are carnivores that hunters reported having no use and are left at the site of kill when captured or slaughtered (Table 1). Most hunters (*N* = 33 hunters, 70.2%) declared to have killed wild animals (e.g., snakes and carnivores) to protect their dogs during the hunt.

All informants reported that dogs captured and/or killed wild animals without distinction between the prey’s sex and age. When asked how they proceeded when capturing pregnant females and/or cubs, 22 (46.8%) hunters stated that they release them back to nature, 11 (23.4%) release the female and raise the cubs at home, 11 (23.4%) take the female and cubs home, and 3 (6.4%) kill them or take them home. Hunters mentioned that they raise the cubs at home and later use them as a stimulus to train their dogs for hunting.

All species cited are included in the Red List of the International Union for Conservation of Nature and the Brazilian List of Fauna Threatened with Extinction. Most species cited (*N* = 15) are listed as Least Concerned in both lists. Four species (all nontarget prey) are listed as Vulnerable in at least one list (*Puma yagouaroundi, Leopardus tigrinus, Leopardus wiedii,* and *Kerodon rupestris*). *Leopardus tigrinus* and *Leopardus wiedii* are also listed as Endangered and Near Threatened, respectively (Table 1).

### Hunters’ perceptions of hunting with dogs

The informants perceived that the number of specimens hunted with dogs per expedition ranged from 1 to 7 (mean ± SD = 3 ± 1.5 specimens), and the perceived number of specimens hunted without dogs (i.e., using traps) per expedition ranged from 0 to 2 (mean ± SD = 0.9 ± 0.4 specimens). According to the Wilcoxon test, hunting with dogs was perceived as more efficient than hunting without dogs (*W* = 1128, *p* < 0.001).

For hunters, the success of the expedition depends mostly on the hunting location (*N* = 23), the dogs’ training/experience (*N* = 23), luck (*N* = 24), and fewer (*N* = 4) responded that also depends on the moon (full moon, dark moon). According to their perception, the target species hunted in greatest quantity per expedition are *Euphractus sexcinctus* (from 2 to 6, *N* = 43), and *Conepatus semistriatus* (from 2 to 6, *N* = 7) because “there’s more and are easier to capture.” *Euphractus sexcinctus* was mentioned by all hunters to be the “easiest animal to be found in the area with hunting dogs.” In turn, the hardest target species to be found in the area with hunting dogs were *Tamandua tetradactyla* (*N* = 39), and *Dasypus novemcinctus* (*N* = 30). To most hunters (*N* = 37), the abundance of these animals is low or very low because “there are many hunters” and “some hunters do not respect the reproductive period” (i.e., the season of the year a species reproduces, when there is the presence of pregnant females and/or cubs).

## Discussion

In the present study, we found that hunters that hunted with dog assistance were mostly men, of different ages, that have an occupation in agriculture, receive less than the Brazilian minimum wage, and had non or a low level of formal education. The socioeconomic profile of hunters found in this study is in agreement with other studies conducted in Brazil [20, 38–41]. It is expected that factors such as gender, age, occupation, income, and access to formal education can influence the type of activities related to and the use of natural resources [42–44]. In traditional communities, hunting is reported to be almost exclusively a male activity [but see 45, 46]. Research suggested that in hunter-gatherer societies the sexual division of labor with males as hunters and females as gatherers was an ancestral pattern related to the constraints faced by females’ pregnancy and childcare [47]. However, recent findings indicate that this male-biased behavior is a recent cultural motivation [48]. Interestingly, some studies have reported that females can play important roles when hunting with dogs in the Neotropics [see 49]. Most hunters’ occupation in agriculture, their low income, and low formal educational level found here are in accordance with the need for an alternative subsistence and food source in the challenging environment of the Caatinga [20, 21]. Moreover, in this region, hunting activities have a cultural importance that has been practiced for a long time [1]. The knowledge and practices of this activity are passed down between generations, starting in early childhood, and can be maintained throughout life, explaining the fact that we found hunters of the most diverse ages [20].

As part of this practice, hunters typically train their hunting dogs by themselves [20, 49, 50]. The training usually consists in taking younger dogs on hunts so that they can learn, by imitation, from older and more experienced dogs [20, 49, 51, 52]. In addition, dogs can be trained by exposing them to wild animals reared at home (e.g., armadillos, as reported here) to stimulate their senses and natural tendency to hunt [20, 50]. Overall, when hunting with dogs, all hunters preferred to hunt using mixed-breed dogs, as found in other Neotropical areas (e.g., indigenous and traditional Amazonian communities: 49, 53). We found that most hunters hunt with multiple dogs (up to 3), which is a practice also described in other studies [24, 49]. By taking several dogs, hunters can track larger areas and increase the probability of successfully detecting and capturing wild animals [21, 54]. Regarding hunting dogs’ sex, there was no clear preference among hunters, with some preferring males, others preferring females, and others having no preference at all. It seems that these preferences are more related to the hunter’s personal perceptions and experiences than to hunting efficiency influenced by dogs’ sex. Overall, it appears that when a dog is well-trained, both sexes are used and perceived as good, efficient hunters.

The motivations for hunting with dogs were mainly associated with subsistence (for food/flavor) and entertainment (for sport/pleasure), few hunters also mentioned they hunted for trade, medicinal purposes, and eventually, to protect their dogs against dangerous animals. This is in accordance with the findings of other hunting studies conducted in the semiarid region and other parts of the world [15, 39, 50, 55]. Specifically, the hunting of target species with the help of dogs seems to be a recreational activity among hunters, in which the main motivations are entertainment and the appreciation of the bushmeat flavor. Indeed, taste preference can increase the chances of a species being killed [56]. Therefore, besides the relevant subsistence role of hunting in the semiarid, hunting with dogs seems to also have an important cultural role among hunters, playing a significant part in their social life. The most salient target prey species were terrestrial mammals hunted at night (i.e., *Euphractus sexcinctus*, *Conepatus semistriatus*, *Dasypus novemcinctus*, and *Tamandua tetradactyla*). These species are reported to also be hunted using other strategies/techniques such as tracking and traps [20], and some hunters associate tools or weapons to get easier access and kill the prey. Most of these species seek refuge (in burrows or trees) or assume a defensive posture when pursued by dogs, which makes them particularly vulnerable to this practice [49].

Only two reptile species were reported to be hunted with the help of dogs in Taperoá and Salgadinho, *Salvator merianae,* and *Iguana iguana*. Both are hunted as a food resource, and the first one has also known medicinal uses for several diseases [57, 58]. Other hunting studies in the region showed that game fauna is more represented by bird species, followed by mammals and reptiles [39, 59]. Besides the food motivation, another strong motivation to capture birds is the pet market. People capture and keep birds because of their beautiful colors and songs [60]. However, when hunting with dogs, most birds are quickly frightened/scared and fly away. Here, we found four bird species hunted with the assistance of dogs (*Crypturellus* sp., *Cariama cristata*, *Dendrocygna viduata*, and *Nothura boraquira*). *Crypturellus* sp. has been reported elsewhere to be a species hunted with the help of trained dogs that flush the birds so that the hunter can shoot it as it takes flight [20]. For other species, such as *Dendrocygna viduata*, dogs push the birds into the forest, which facilitates the capture [61]. Hunting with dogs usually results in the death of prey, all species of birds hunted with dogs in the surveyed area are used as food.

Hunters perceived that the number of specimens hunted per expedition when hunting with dogs could be three times higher than when hunting without dogs. Some studies showed that hunting with dogs provides a higher return, especially when combined with other tools such as shotguns, bows, or other accessories [49, 62–64]. From an optimal foraging perspective, hunting with dogs has both costs and benefits, involving a trade-off between increased encounter rates of several profitable prey species and time costs related to longer pursuits of prey, when compared with hunting only with guns [62]. For example, in Nicaragua, hunting with dogs was profitable in encountering eight times more agoutis (*Dasyprocta punctata*) than hunting without dogs [62]. However, the efficiency of hunting with dogs can largely vary between locations, as it also depends on other variables such as the prey’s size (energetic benefit) and vulnerability (antipredator behavior/strategies), hunter’s ability to understand dog signs [e.g., 65], and dog’s ability to track and chase prey [24, 49, 53, 66].

Hunting with dogs can be more efficient at providing meat, but also at killing threatened species [24]. Dogs optimize hunting success and select a diversity of species that are more resilient to hunting [53]. However, this activity can have a larger impact on wildlife than hunting without dogs, especially when complemented with other technologies such as firearms [62, 67, 68]. Even though most hunters reported that they maintain their dogs restricted in their household, half of them mentioned that at some point, the dog released itself and escaped to forest areas. In addition, some hunters take their dogs for companionship during their daily activities, typically unrestrained and free to roam. Due to their unselective foraging behavior and self-sufficiency for hunting, these dogs have ended up capturing wild animals by themselves when not on hunting expeditions. This can cause higher pressure on the local fauna in several ways.

First, hunting dogs can track and hunt wild animals not showing any selection on the prey, including juvenile animals, females with cubs, and nontarget species that hunters would not normally pursue (e.g., inedible threatened species) [24]. Indeed, all hunters reported that their dogs capture wild animals without discrimination between the prey’s sex and age, which can have negative consequences at the game population level/dynamics (maintenance or growth), especially in long-lived, slow-reproducing/breeding species (low reproductive rates) [24]. Moreover, hunters reported killing some species for the sole purpose of protecting their dogs, resulting in the killing of carnivores such as *Cerdocyon thous*, *Didelphis albiventris*, *Procyon cancrivorus*, *Galictis vittata*, *Herpailurus yagouaroundi, Leopardus tigrinus*, and *Leopardus wiedii* that are left at the site of the kill. The last three species are classified as vulnerable or endangered species [34, 35]. Second, unrestrained dogs can get lost and become feral dogs, which survive and reproduce independently of human assistance, can become aggressive towards humans, travel at packs, and acquire their primary subsistence by hunting or scavenging like other wild canids [69]. Furthermore, feral dogs can transmit diseases to wild animals, especially carnivores (e.g., distemper, rabies, parvovirus) [70, 71], and affect the habitat use and ranging behavior of some species [72, 73]. Finally, if not neutered, unrestrained dogs can reproduce with other free-roaming/feral dogs and perpetuate all problems mentioned above.

The socioeconomic reality of hunters might be a limiting factor for different aspects of hunting dogs’ keeping practices found in this study. For example, a low monthly income and a low educational level can prevent hunters from offering proper housing, diet, and health care to their dogs due to insufficient financial resources and/or lack of information. In turn, this can compromise dogs’ welfare and sanitary conditions, resulting in injuries, malnutrition, as well as the proliferation of parasites, pathogens, and diseases causing unhealthiness in dogs and representing a potential public health risk also affecting humans and wildlife [74]. It is worth noticing that most hunters vaccinated their dogs in free rabies campaigns, indicating the importance of implementing government actions, not only large-scale dog mass vaccination and neutering programs, but also actions promoting responsible animal ownership to improve information and attitudes toward dogs, their maintenance and care.

Understanding the context in which humans and wildlife interact is critical to the establishment of successful conservation strategies. Given the high degree of human dependence on natural resources, it is important to take into account both conservation and human survival. Therefore, conservation strategies must try to reconcile both needs. Overall, we found that hunting with dogs represents a complex set of local variables, including characteristics of dogs and prey species, hunters’ motivations, strategies, and complementary technologies that should be considered according to each particular situation. Considering the socioeconomic and ecological realities of the semiarid region, the Caatinga, hunters should be included in wildlife management debates aiming to formulate conservation plans focusing on regulating hunting activities (with and without dogs) to mitigate the threat to game species that are more susceptible to over-hunting, while allowing sustainable hunting practices. Management actions could include selective harvesting by sex and age, limiting the harvests of females and cubs; seasonal hunting restrictions during the reproductive seasons of certain species; and the establishment of hunting quotas, restricting which game species and the number of specimens that can be hunted per hunting season. In addition, actions related to the proper maintenance and care of hunting dogs, which are valuable contributors to household subsistence and livelihoods, must be taken into account to prevent additional threats to local fauna. For example, population management practices and public policies aimed at veterinary care to prevent undesired reproduction and the spread of diseases (e.g., castration and vaccination campaigns); limiting dogs from having access to the forest by themselves; and the education of owners about responsible ownership. Finally, adequate supervision for compliance with these management actions is crucial, as the goal would not be the depletion of this cultural activity so deeply rooted in the life of human communities, but to avoid local or functional extinction of animal populations or species.

## Conclusion

In the Brazilian semiarid region, men of different ages, with low levels of formal education, that have occupations in agriculture and receive less than a minimum wage, hunt with the assistance of dogs. Hunters perceived that hunting with dogs is three times more efficient, in terms of the number of specimens hunted per expedition, than hunting without dogs. Their main motivations are associated with subsistence and entertainment purposes. Hunters used mainly well-trained mixed-breed dogs targeting terrestrial mammals at night. However, they end up hunting several nontarget species, of which some are used as food or for medicinal purposes, while others are left at the site of the kill. All game species reported here are of conservation concern and included in diverse categories of IUCN and Brazilian Red Lists of Threatened Species. Investigating the set of local variables and patterns that characterize and motivate hunting with dogs’ activities is crucial to understand the context in which humans and wildlife interact, as well as its impacts on game species and human subsistence. This information should be used to implement successful wildlife conservation strategies, without ignoring specific local communities’ needs.

## Data Availability

The datasets used and/or analyzed during the current study are available from the corresponding author upon reasonable request.

## Abbreviations

BSh: Hot semiarid (steppe) climate
°C: Degrees celsius
mm: Millimeters
SD: Standard deviation
R$: Brazilian reais
US$: United States dollar
LC: Least concerned
VU: Vulnerable
EN: Endangered
NT: Near threatened
NS: No school
IES: Incomplete elementary school
CHS: Complete high school
IHE: Incomplete higher education
CHE: Complete higher education
